# Inequalities in the social determinants of health of Aboriginal and Torres Strait Islander People: a cross-sectional population-based study in the Australian state of Victoria

**DOI:** 10.1186/s12939-014-0091-5

**Published:** 2014-10-18

**Authors:** Alison Markwick, Zahid Ansari, Mary Sullivan, Lorraine Parsons, John McNeil

**Affiliations:** Department of Health, Health Intelligence Unit, Prevention and Population Health Branch, 50 Lonsdale Street, Melbourne, 3000 Victoria Australia; Department of Health, Aboriginal Health Branch, 50 Lonsdale Street, Melbourne, 3000 Victoria Australia; Department of Epidemiology and Preventive Medicine, School of Public Health and Preventive Medicine, Monash University, 99 Commercial Rd, Melbourne, 3004 Victoria Australia

## Abstract

**Introduction:**

Aboriginal Australians are a culturally, linguistically and experientially diverse population, for whom national statistics may mask important geographic differences in their health and the determinants of their health. We sought to identify the determinants of health of Aboriginal adults who lived in the state of Victoria, compared with their non-Aboriginal counterparts.

**Methods:**

We obtained data from the 2008 Victorian Population Health Survey: a cross-sectional computer-assisted telephone interview survey of 34,168 randomly selected adults. The data included measures of the social determinants of health (socioeconomic status (SES), psychosocial risk factors, and social capital), lifestyle risk factors, health care service use, and health outcomes. We calculated prevalence ratios (PR) using a generalised linear model with a log link function and binomial distribution; adjusted for age and sex.

**Results:**

Aboriginal Victorians had a higher prevalence of self-rated fair or poor health, cancer, depression and anxiety, and asthma; most notably depression and anxiety (PR = 1.7, 95% CI; 1.4–2.2). Determinants that were statistically significantly different between Aboriginal and non-Aboriginal Victorians included: a higher prevalence of psychosocial risk factors (psychological distress, food insecurity and financial stress); lower SES (not being employed and low income); lower social capital (neighbourhood tenure of less than one year, inability to get help from family, didn’t feel valued by society, didn’t agree most people could be trusted, not a member of a community group); and a higher prevalence of lifestyle risk factors (smoking, obesity and inadequate fruit intake). A higher proportion of Aboriginal Victorians sought help for a mental health related problem and had had a blood pressure check in the previous two years.

**Conclusions:**

We identified inequalities in health between Aboriginal and non-Aboriginal Victorians, most notably in the prevalence of depression and anxiety, and the social determinants of health (psychosocial risk factors, SES, and social capital). This has implications for evidence-based policy development and may inform the development of public health interventions.

## Introduction

Inequalities in health between Aboriginal and Torres Strait Islander people and their non-Aboriginal and Torres Strait Islander counterparts are noted by the World Health Organization (WHO) to be the largest in the world [1]. Nationally, life expectancy for the Aboriginal and Torres Strait Islander population born in 2010–2012, was estimated to be 10.6 years lower than that of the non-Aboriginal and Torres Strait Islander population in males and 9.5 years lower in females [2]. Non-communicable diseases are responsible for 70% of the health gap, leading with cardiovascular disease (23%), followed by diabetes (12%), mental disorders (12%) and chronic respiratory diseases (9%) [3].

Understanding the historical reasons for the ongoing health inequalities of Aboriginal and Torres Strait Islander health is critical in gaining the awareness to successfully engage with Aboriginal and Torres Strait Islander people and together envisaging a way forward. With the colonisation of Australia, the annihilation of the Aboriginal and Torres Strait Islander people began, through widespread massacres and the introduction of previously unknown infectious diseases. By 1850 only 10% of the Aboriginal and Torres Strait Islander population remained alive [4]. Aboriginal and Torres Strait Islander people were dispossessed of their lands and subsequently segregated onto reserves or missions. Government assimilation policies oversaw the widespread destruction of families and communities through the removal of their children, commonly referred to as the ‘stolen generations’ [5]. As Tom Calma, former Aboriginal Social Justice Commissioner stated; *“Indigenous peoples are not merely ‘disadvantaged citizens’. The poverty and inequality that they experience is a contemporary reflection of their historical treatment as peoples. The inequality in health status that they continue to experience can be linked to systemic discrimination”* [6].

Tom Calma went on to say that recognising the contemporary impact of colonisation on Aboriginal and Torres Strait Islander people remains a major challenge for those who seek to understand the determinants of health among Aboriginal and Torres Strait Islander communities. Essentially, colonisation created significant barriers towards improving the health of Aboriginal and Torres Strait Islander people, and these barriers work on many levels; physician-patient interaction, delivery of health services as a whole, and the wider political and economic stage. Strategies and interventions need to be implemented at each of these levels to help create a holistic and culturally sensitive approach towards improving the health of Aboriginal and Torres Strait Islander people.

At the level of health care, it is necessary to broaden our definitions of health to include the physical, mental, and spiritual wellbeing of entire communities, not just the symptomatic treatment of the individual. Western biomedical models of health, with their predominant focus on diagnosis, treatment and prevention, have the effect of reducing the Aboriginal and Torres Strait Islander identity to a series of health problems that need fixing. The constant discourse over Aboriginal and Torres Strait Islander dysfunction and inadequacy in public health practice; “disconnects Aboriginal and Torres Strait Islander people from their own identities, in a manner similar to past oppressive policies of colonisation, assimilation and integration” [7]. Moreover, the dominance of the biomedical model has resulted in public health efforts predominately directed at addressing the lifestyle risk factors on a platform of “personal responsibility”; mainly through health education. We believe that this reinforces and perpetuates prejudice and racism; a key determinant of ill-health in Aboriginal and Torres Strait Islander people. Thus, health education in this form is disempowering for Aboriginal and Torres Strait Islander people and reinforces existing feelings of low self-esteem [7,8].

The Aboriginal and Torres Strait Islander construct of health is not just about the physical wellbeing of the individual. It is the social, emotional and cultural wellbeing of the entire community, a concept that is usually ignored by mainstream health services. It is therefore unsurprising that mainstream health services face additional challenges in trying to gain the trust of Aboriginal and Torres Strait Islander people. In terms of health service delivery, Aboriginal and Torres Strait Islander community controlled health services emphasise the importance of a holistic approach towards Aboriginal and Torres Strait Islander health care, where physical and mental wellbeing is linked to its historical and cultural context. They are also particularly vocal in deploring the lack of time spent on Aboriginal and Torres Strait Islander studies in medical curriculums and are taking the initiative to educate non-Aboriginal and Torres Strait Islander doctors working with their organizations [9].

However, as Aboriginal and Torres Strait Islander Australians are a culturally, linguistically and experientially diverse population, national statistics may mask important geographic differences in their health and the determinants of their health. While the state of Victoria has the second largest population in the country, it has the lowest proportion of Aboriginal and Torres Strait Islander people [10]. In 2011, approximately 47,000 Victorians self-identified as being of Aboriginal and Torres Strait Islander origin; representing 7% of the Australian Aboriginal population and 0.9% of the total Victorian population [10]. There is a paucity of data on the health of Aboriginal and Torres Strait Islander people in Victoria and what is available is often of poor quality.

The Victorian Population Health Survey (VPHS), conducted annually since 2001 with a sample size of approximately 7,500, typically only recruited 50 to 60 people per survey who identified as Aboriginal or Torres Strait Islander; a sample too small to provide reliable estimates for most of the determinants and outcomes investigated. However, in 2008, the total sample size was increased to approximately 34,000 in order to be able to estimate prevalence at the level of the local government area (LGA). Fortuitously, the larger sample also included 339 Aboriginal and Torres Strait Islander people, a sample that permitted the reliable estimation of the prevalence of most of the determinants and outcomes investigated. Consequently we were able, for the first time, to report on the population health of Aboriginal and Torres Strait Islander people in Victoria [11].

The purpose of this paper is to expand on the initial analysis of the 2008 VPHS data, in order to identify the significant gaps in the determinants of health of Aboriginal and Torres Strait Islander people in Victoria. The analysis was based on a public health model of the social determinants of health, which was used to inform the development of the VPHS [12]. It is important to note however, that the model is based on a western concept of the social determinants of health and therefore precludes information on some of the key determinants of Aboriginal and Torres Strait Islander health. The model posits that the social determinants (socioeconomic, psychosocial risk factors, and community and societal characteristics) impact directly, and indirectly, on the health status of the population via lifestyle risk factors (referred to as disease-inducing behaviours in the model) and access to, and/or use of the health care system. We present data for each of the domains of the model. Data collected by the VPHS for the domain of ‘community and societal characteristics’ were primarily indicators of social capital and will be referred to as such hereon.

Please note, that from this point forward, for ease of reading, the term Aboriginal will be used to refer to Aboriginal and Torres Strait Islander peoples, but not to undermine their respective distinct identities.

## Methods

### Data source, sampling frame and sample size

Data were collected as part of the VPHS in 2008, a state-wide computer-assisted telephone interview survey of a randomly selected sample of adults, aged 18 years or older, who resided in private dwellings in Victoria and had access to a landline telephone. The sampling frame was an electronic listing of Victorian telephone exchange prefixes and localities. Random digit dialling was used to generate a sample of telephone numbers that formed the household sample. Only one person aged 18 years or older, per household, with the most recent birthday, was selected for interview. The sample was stratified by LGA; with a target sample of 426 individuals per LGA. The total sample achieved was 34,168 adults, including 339 Aboriginal respondents. The response rate, defined as the proportion of households where contact was made and an interview completed, was 65 per cent.

### Weighting

In order to control for participation bias, the survey data were weighted to reflect the age/sex/geographic distribution of the estimated resident population of Victoria, together with the probability of selection of the household, and respondent within the household. The data was not weighted by ethnicity as the purpose of the survey was to provide prevalence estimates for the Victorian population at the LGA level.

### Ethics statement

The Department of Health Human Research Ethics Committee approved the survey in accordance with the guidelines of the Declaration of Helsinki. We did not initially refer to the National Health and Medical Research Council (NHMRC) Values and Ethics - Guidelines for Ethical conduct in Aboriginal and Torres Strait Islander Health Research. as the survey was not conducted specifically to answer a research question about the Aboriginal population [13]. Data on Aboriginal status and ethnicity were collected as demographic variables at the end of the interview, for the purpose of determining how closely the survey sample matched the true Victorian population. It was entirely fortuitous that we recruited a sufficient number of respondents who identified as Aboriginal, to enable this work. Moreover, it is in the nature of CATI surveys that they are conducted anonymously and therefore consent is not specifically obtained from each respondent other than the respondent’s verbal agreement to participate in the interview. However, we ensured that the presentation and interpretation of the data was done in ways to avoid harm to Aboriginal people, by consulting and collaborating with the Aboriginal Health Branch in the Department of Health, who have strong links with key Aboriginal organisations such as the Victorian Aboriginal Community Controlled Health Organisation and its component organisations, and the Onemda VicHealth Koori Health unit at the University of Melbourne. This was consistent with the principles of the NHMRC Values and Ethics - Guidelines for Ethical Conduct in Aboriginal and Torres Strait Islander Health Research.

### Statistical analysis

We analysed the survey data using the Stata statistical software package version 12 [14]. We calculated prevalence ratios (PR) using a generalised linear model with a log link function and binomial distribution, and adjusted for age and sex [15]. We accepted statistical significance at the p < 0.05 level.

### Coding of variables

Sex was a binary variable and 0 = female (referent group) and 1 = male. Age was stratified by 10-year age groups, where category 1 = 18–29 years (referent group), 2 = 30–39 years, 3 = 40–49 years, 4 = 50–59 years, 5 = 60–69 years, 6 = 70 years and older. All other binary variables were coded as 0 = No (referent group) and 1 = yes.

### Variables

We determined Aboriginal status by asking: “Are you of Aboriginal or Torres Strait Islander origin?” Respondents who stated that they were Aboriginal (n = 258), Torres Strait Islander (n = 40) or both (n = 41) were combined and a binary variable created. Only 0.3% of the total sample declined to answer or stated that they did not know, and were coded as missing data.

Indicators of SES included total annual household income (HI) and employment status. We created six binary variables; (1) HI less than $80,000, (2) HI less than $40,000, (3) HI less than $20,000, (4) unemployed, (5) not in the labour force, and (6) unable to work. Household income was defined as total income before tax from all sources including social security payments, child support, and investments over the previous 12 months.

Indicators of psychosocial risk factors included food insecurity, psychological distress and financial stress. We determined that a respondent was food insecure if they answered in the affirmative the following question “In the last 12 months, were there any times that you ran out of food, and couldn’t afford to buy more?” We used the Kessler 10 Psychological Distress Scale (K10) to determine the respondent’s level of psychological distress level in the four weeks preceding the survey [16]. We determined financial stress by asking if, in an emergency, the respondent could raise $2,000 within two days. We created four binary variables: (1) food insecure, (2) very high psychological distress (K10 score ≥30), (3) high/very high psychological distress (K10 ≥ 22), and (4) unable to raise $2,000.

We assessed the respondent’s social environment by asking about the length of time lived in their neighbourhood (area of residence) and created a binary variable: lived in neighbourhood for less than one year. We determined the respondent’s social and support networks by asking a series of questions: whether they could get help from family, neighbours or friends if needed; if they had attended any support group meetings over the last two years; and if they had access to community services and resources such as libraries, maternal and child health centres, and neighbourhood centres when needed. We created five binary variables: unable to get help from (1) family, (2) neighbours and/or (3) friends, (4) attended a support group meeting, and (5) unable to get access to community services and resources. We measured the level of social and civic trust by asking the respondent two questions, respectively: “Do you agree that most people can be trusted?”, and “Do you feel valued by society?” We created two binary variables: (1) did not believe that most people could not be trusted (social trust) and (2) did not feel valued by society (civic trust). We determined the level of community and civic engagement by asking the respondent if they were a member of one or more of the following groups: a sports, church, school, professional, and/or other community group. We created a binary variable; not a member of any group.

The lifestyle risk factors that we investigated included: excessive consumption of alcohol; overweight and obesity; smoking; inadequate physical activity; inadequate fruit and vegetable consumption; and hypertension. We determined excessive alcohol consumption by comparing the respondent’s pattern of alcohol consumption with the *2001 Australian recommended guidelines* [17]. If a respondent exceeded the recommended threshold level of alcohol consumption deemed to be safe, they were classified as engaging in excessive alcohol consumption. We created three binary variables of excessive alcohol consumption based on frequency of occurrence: (1) at least once a year, (2) at least once a month, and (3) at least once a week. We calculated body mass index (BMI) based on the respondent’s self-reported height and weight, and categorised body weight status according to the recommendations of the WHO: overweight = BMI 25.0–29.9 kg/m^2^ and obesity = BMI ≥30 kg/m^2^ [18]. We created two binary variables: (1) overweight or obese and (2) obese. We categorised a respondent as being a smoker if they reported smoking daily or occasionally. We measured a respondent’s physical activity level by asking a series of questions about their usual physical activity over the course of a week. We categorised the respondent’s level of physical activity according to the *1999 National Physical Activity Guidelines for Australians* and created a binary variable: inadequate physical activity, defined as less than 150 minutes of physical activity in total or ≥150 minutes but fewer than 5 sessions per week [19]. We determined a respondent’s daily fruit and vegetable intake by asking the respondent to report the average number of serves of fruit and vegetable they usually consumed. We categorised the respondent’s intake according to the *2003 Dietary guidelines for Australian adults*, and created two binary variables: (1) inadequate fruit intake (defined as less than 2 serves per day) and (2) inadequate vegetable intake (defined as less than 5 serves per day) [20]. We determined a respondent to be hypertensive by asking if the respondent had ever been diagnosed by a doctor with high blood pressure.

We measured the use of health care services by asking respondents a series of questions which included: whether a health professional had performed a check on their blood pressure, cholesterol and/or blood glucose in the previous two years, if they had attended a public hospital in the previous year, if they had consulted with a health professional for a mental health related problem in the previous year, if they had ever seen an eye health professional, and if they had had a test to detect bowel cancer in the previous two years. We created binary variables for each service. The services represented were chosen to meet the needs of various survey stakeholders. One of the internal stakeholders was the Aboriginal Health Branch in the Department of Health, with strong links to representative organisations of the Aboriginal community in Victoria.

Self-reported health status has been shown to be a reliable predictor of ill-health, future health care use and subsequent mortality [21]. Therefore, we assessed the overall health status of the respondent by asking them to rate their general health as: excellent, very good, good, fair, or poor. We created a binary variable: fair or poor self-reported health. We also asked respondents if they had ever been diagnosed by a doctor with one of the following diseases or conditions: cancer, depression and/or anxiety, asthma (past and in the preceding 12 months), and arthritis. The diseases and conditions were chosen as they represent the national health priority areas of Australia. While data on type 2 diabetes, stroke, and heart disease was also collected, the sample of Aboriginal people reporting these was too small to analyse.

### Missing data

Less than 2% of respondents refused to answer or were unable to answer the survey questions for all variables with the exception of total annual household income (18%), BMI (6%), physical activity (5%), psychological distress (4%), the ability to get help from neighbours (3%), and ‘did not feel valued by society’ (6%). Missing data was excluded from the analysis. The models were rerun with the missing data included in the denominator, but this made negligible difference to any of the results.

## Results

### Summary

We observed statistically significant differences between Aboriginal and non-Aboriginal Victorians in 14 of the 19 social determinant variables (Table 1) and three of the 7 lifestyle risk factors investigated (Table 2). The greatest effect size was for two of the 4 psychosocial risk factors; very high levels of psychological distress (PR = 4.4; p < 0.001) and food insecurity (PR = 3.4; p < 0.001). This was followed in decreasing order: unable to work, unemployed, unable to get help from family when needed, high/very high psychological distress, and lived in the neighbourhood less than one year, all social determinants with prevalence ratios ≥2.0 and <3.0. Thereafter, all statistically significant prevalence ratios were less than 2.0 in the following descending order: financial stress, smoker, income of less than $20,000, did not feel valued by society, obese, income of less than $40,000, did not believe that most people could be trusted, inadequate fruit intake, not a member of a group, and an income of less than $80,000.

**Table 1:** **Age and sex-standardised prevalence, prevalence ratios and 95% confidence intervals (CI) of social determinants of health, by Aboriginal status**

**Indicator**	**Prevalence (%)**			**Prevalence ratio (95% CI)**		
	**Age-adjusted**		**Crude**			
	**Non-Aboriginal**	**Aboriginal**	**Aboriginal**	**Non-Aboriginal**	**Aboriginal (95% CI)**	**p value**
**Socioeconomic determinants**						
Income less than $80,000	63.7	81.1	79.6	1.00	1.2 (1.1-1.3)	<0.001
Income less than $40,000	32.4	48.4	44.1	1.00	1.4 (1.2-1.7)	<0.001
Income less than $20,000	12.4	18.9	17.5	1.00	1.6 (1.1-2.3)	0.014
Unemployed	3.6	8.7	10.2	1.00	2.4 (1.4-4.2)	0.001
Not in labour force	35.5	39.2	33.9	1.00	1.1 (0.9-1.3)	0.444
Unable to work	2.9	7.3	6.8	1.00	2.5 (1.6-4.0)	<0.001
**Psychosocial risk factors**						
Food insecure	5.4	17.7	20.3	1.00	3.4 (2.3-5.1)	<0.001
High/very high psychological distress^a^	11.6	22.6	25.3	1.00	2.1 (1.5-2.9)	<0.001
Very high psychological distress^b^	3.1	11.3	13.7	1.00	4.4 (2.6-7.4)	<0.001
Financial stress^c^	11.8	19.4	20.7	1.00	1.7 (1.2-2.4)	0.002
**Social capital**						
Lived in neighbourhood less than 1 year	8.3	14.8	19.4	1.00	2.0 (1.3-3.0)	0.001
Unable to get help from family	7.2	15.2	15.0	1.00	2.1 (1.4-3.2)	<0.001
Unable to get help from neighbours	25.4	26.8	28.8	1.00	1.1 (0.8-1.4)	0.758
Unable to get help from friends	5.0	3.0	3.1	1.00	0.7 (0.4-1.3)	0.231
Attended support group meeting	10.0	14.5	13.7	1.00	1.4 (1.0-2.1)	0.084
Unable to access community services or resources	13.5	13.7	14.9	1.00	1.1 (0.7-1.7)	0.626
Did not believe most people could be trusted (social trust)	20.9	27.3	29.2	1.00	1.4 (1.0-1.8)	0.035
Did not feel valued by society (civic trust)	13.2	18.9	20.1	1.00	1.6 (1.1-2.3)	0.027
NOT a member of a group^d^	38.9	47.6	49.1	1.00	1.3 (1.0-1.5)	0.016

^a^Kessler Psychological Distress Scale (K10) score ≥22.

^b^K10 score ≥30.

^c^Unable to raise $2,000 within two days in an emergency.

^d^Sports, church, school, professional or other community action group.

**Table 2:** **Age and sex-standardised prevalence, prevalence ratios and 95% confidence intervals (CI) of lifestyle risk factors, by Aboriginal status**

**Risk factor**	**Prevalence (%)**			**Prevalence ratio (95% CI)**		
	**Age-adjusted**		**Crude**			
	**Non-Aboriginal**	**Aboriginal**	**Aboriginal**	**Non-Aboriginal**	**Aboriginal (95% CI)**	**p value**
Risky drinker^a^ at least yearly	45.7	44.3	50.7	1.00	1.0 (0.9-1.2)	0.789
Risky drinker at least monthly	31.1	29.7	31.8	1.00	1.0 (0.7-1.3)	0.769
Risky drinker at least weekly	17.6	15.7	16.9	1.00	0.9 (0.6-1.6)	0.788
Obese^b^	17.6	24.7	24.6	1.00	1.5 (1.1-2.0)	0.020
Overweight or obese^c^	51.5	57.2	53.7	1.00	1.1 (0.9-1.2)	0.395
Smoker	19.1	30.4	33.9	1.00	1.6 (1.2-2.1)	<0.001
Inadequate physical activity	34.3	35.8	33.9	1.00	1.1 (0.8-1.3)	0.696
Inadequate fruit intake	51.5	63.5	66.1	1.00	1.3 (1.1-1.5)	<0.001
Inadequate vegetable intake	91.8	90.4	90.1	1.00	1.0 (0.9-1.0)	0.153
Hypertensive	26.0	25.6	21.5	1.00	1.0 (0.8-1.3)	0.971

^a^At short-term risk of alcohol-related harm.

^b^Body mass index (BMI) ≥30 kg/m^2^.

^c^BMI ≥25 kg/m2.

### Socioeconomic status

Aboriginal Victorians were significantly more likely than their non-Aboriginal counterparts, to have lower household incomes, be unemployed, and unable to work (Table 1).

### Psychosocial risk factors

Aboriginal Victorians had a significantly higher prevalence of all psychosocial risk factors: psychological distress, food insecurity, and financial stress (Table 1).

### Social capital

Aboriginal Victorians were significantly more likely than their non-Aboriginal counterparts, to have lived in their neighbourhood for less than one year, be unable to get help from family, have low levels of social and civic trust, and not be a member of a community group (Table 1). By contrast, Aboriginal Victorians were no more or less likely as non-Aboriginal Victorians to be unable to get help from friends and/or neighbours, to attend a support group, and to be unable to access community services and resources.

### Lifestyle risk factors

Aboriginal Victorians had a significantly higher prevalence of obesity, smoking, and an inadequate fruit intake (Table 2). By contrast, there were no significant differences between Aboriginal and non-Aboriginal Victorians in the prevalence of excessive alcohol consumption, inadequate physical activity and/or vegetable intake, and hypertension.

### Health care

There were no significant differences in health care service use between Aboriginal and non-Aboriginal Victorians, with the exception that Aboriginal Victorians were significantly more likely to have had a blood pressure check, and/or to have sought help from a health professional for a mental health related problem (Table 3).

**Table 3:** **Age and sex-standardised prevalence, prevalence ratios and 95% confidence intervals (CI) of health care service use, by Aboriginal status**

**Health care service**	**Prevalence (%)**			**Prevalence ratio (95% CI)**		
	**Age-adjusted**		**Crude**			
	**Non-Aboriginal**	**Aboriginal**	**Aboriginal**	**Non-Aboriginal**	**Aboriginal (95% CI)**	**p value**
Had a blood pressure check^a^	79.6	83.0	79.3	1.00	1.0 (1.0-1.1)	0.001
Had a blood cholesterol check^a^	57.2	59.4	52.1	1.00	1.0 (0.9-1.1)	0.459
Had a blood glucose check^a^	53.6	56.5	50.9	1.00	1.1 (0.9-1.2)	0.457
Attended a public hospital^b^	47.1	50.3	52.0	1.00	1.1 (0.9-1.3)	0.372
Saw professional for mental health^b^	11.3	19.2	20.0	1.00	1.7 (1.1-2.4)	0.008
Ever saw an eye health professional	77.6	75.2	69.8	1.00	1.0 (0.9-1.1)	0.975
Bowel examination for bowel cancer^a^	29.7	36.1	34.9	1.00	1.2 (0.9-1.7)	0.300

^a^In preceding two years.

^b^In preceding 12 months.

### Health outcomes

Aboriginal Victorians were significantly more likely than their non-Aboriginal counterparts, to report being in fair or poor health, and/or to ever have been diagnosed by a doctor with cancer, asthma (past and present), and depression and anxiety (Table 4). Of particular note was the substantially higher prevalence of depression and anxiety, particularly in Aboriginal men with almost 35% having ever been diagnosed, compared with 14.8% of non-Aboriginal men.

**Table 4:** **Age and sex-standardised prevalence, and prevalence ratios and 95% confidence intervals (CI) of health outcomes, by Aboriginal status**

**Health outcome**	**Prevalence (%)**			**Prevalence ratio (95% CI)**		
	**Age-adjusted**		**Crude**			
	**Non-Aboriginal**	**Aboriginal**	**Aboriginal**	**Non-Aboriginal**	**Aboriginal (95% CI)**	**p value**
Fair or poor self-reported health	18.1	28.0	26.5	1.00	1.5 (1.1-2.1)	0.006
Cancer^a^	6.4	11.8	7.3	1.00	1.7 (1.0-2.7)	0.042
Depression or anxiety^a^	19.7	34.8	35.2	1.00	1.7 (1.4-2.2)	<0.001
Depression or anxiety - males^a^	14.8	34.9	35.7	1.00	2.5 (1.8-3.7)	<0.001
Depression or anxiety - females^a^	24.4	35.7	34.7	1.00	1.4 (1.0-1.9)	0.028
Ever had asthma^a^	21.2	29.3	31.2	1.00	1.4 (1.1-1.8)	0.018
Currently has asthma^b^	10.7	16.4	16.7	1.00	1.6 (1.1-2.3)	0.023
Arthritis^a^	20.1	23.5	17.8	1.00	1.1 (0.9-1.4)	0.342

^a^Ever diagnosed by a doctor.

^b^Diagnosed by a doctor and experienced symptoms in last 12 months.

## Discussion

We observed a higher prevalence of poor health outcomes among Aboriginal Victorians compared with their non-Aboriginal counterparts, consistent with national findings. Aboriginal Victorians had a higher prevalence of all health outcomes investigated, with the exception of arthritis. Of particular note was the higher prevalence of depression and anxiety, where more than one in three Aboriginal men and women in Victoria had been diagnosed with depression and or anxiety, compared with about one in five non-Aboriginal Victorians. This indicates a priority area for intervention and is supported by the finding of a higher prevalence of psychological distress among Aboriginal Victorians, a key risk factor for depression and anxiety, suggesting that preventive interventions are also required. The development of preventive measures will require further research to understand the underlying determinants. For example, prejudice and racism have been shown to be a key determinant of Aboriginal health in Australia, and are associated with high levels of psychological distress [22-24].

In investigating the determinants of health, we found significant disparities between Aboriginal and non-Aboriginal Victorians in fourteen of 19 social determinants and three of 7 lifestyle risk factors. The largest effect sizes were in the difference in prevalence of the psychosocial risk factors of psychological distress, food insecurity and financial stress. Psychological distress impacts negatively on health through a number of pathways both directly and indirectly. In addition to being a risk factor for depression and anxiety, psychological distress has been shown to exacerbate poor health, be a risk factor for the incident development of diseases such as coronary heart disease and stroke, and to increase the engagement in unhealthy behaviours (lifestyle risk factors) [25-27]. In addition to the impact that food insecurity has on nutritional intake, food insecurity also has a range of social and emotional consequences, such as psychological distress, social exclusion, impaired learning and loss of productivity [28].

Our data shows that socioeconomically, Aboriginal Victorians are severely disadvantaged compared with their non-Aboriginal counterparts, with lower household incomes and lower employment rates. While our data supports a socioeconomic explanation of the inequalities in health of Aboriginal Victorians, the study design was cross-sectional and we can therefore make no claims as to causality or its direction. Nevertheless, the weight of scientific evidence supports a socioeconomic explanation of health inequalities, as low SES has been shown to have a significant adverse impact on health status [29,30]. Low household income results in less disposable income to purchase healthy foods, engage in leisure time activities that may be an important source of physical activity, and to afford safe and adequate housing and healthcare. Moreover, a low level of educational attainment puts people at higher risk of unemployment, limits their likelihood of obtaining a job that pays a living wage, and is associated with lower levels of health literacy.

Our data shows significant disparities in social capital between Aboriginal and non-Aboriginal Victorians, with Aboriginal Victorians having lower levels. The preponderance of the evidence shows a clear link between social capital and health outcomes, higher levels of social capital being associated with better health and vice versa [31]. While there is no universally agreed definition of social capital, Bourdieu [32] originally defined social capital as “the aggregate of the actual or potential resources which are linked to possession of a durable network of more or less institutionalised relationships of mutual acquaintance and recognition”. It has been suggested that this definition is more appropriate for Aboriginal peoples given that they continue to be excluded from social networks that could potentially deliver economic and educational benefits [33]. Social capital has been further broken down into three types: bonding, bridging and linking. In the context of Aboriginal communities, bonding social capital refers to relationships within the Aboriginal community, bridging social capital to relationships between the Aboriginal and non-Aboriginal communities, and linking social capital to relationships between the Aboriginal community and the formal and hierarchical institutions of power [34].

It is important to note that social capital is a western concept that has yet to be adapted for the Aboriginal population [33]. Yet it has been suggested that the concept of social capital as defined above may still be a useful tool, particularly in relation to linking social capital [33].

Our data show that Aboriginal Victorians were twice as likely as their non-Aboriginal counterparts to have lived in their neighbourhood for less than one year. Length of neighbourhood tenure is an important indicator of one’s social environment. Frequent relocation impacts adversely on educational opportunities, connection to community and services, and social support networks, reducing all three types of social capital [35,36]. This is consistent with their lower SES, as people on low incomes frequently experience periods of unemployment, and are obliged to relocate frequently in search of jobs and affordable housing.

While we did not find any differences between Aboriginal and non-Aboriginal Victorians in the ability to get help from friends and neighbours, Aboriginal Victorians were significantly less able to get help from family, suggesting a lower level of bonding social capital. One possible explanation for this is that since Aboriginal people in Victoria bore the highest burden of child removal (known as the stolen generations) than any other state in Australia, our finding may reflect the success of past government assimilation policies [37]. Families are an important source of support and lack of family support is likely to increase personal vulnerability, particularly during crises and stressful periods, impacting on health. Our data suggests that Aboriginal Victorians may differ from Aboriginal people who reside in other states, where high levels of bonding social capital have been reported [38]. Moreover, these findings support the development of policies and initiatives to enhance bonding social capital, for example, increased provision of healing centres and programs for the victims of the stolen generations, and support for Aboriginal families.

By contrast, we did not find a difference between Aboriginal and non-Aboriginal Victorians in support group meeting attendance or in the proportion that were able to access community services and resources. However, commensurate with need, it could have been predicted that support group meeting attendance and the proportion that accessed community services and resources would be higher among Aboriginal Victorians. The fact that we did not find this, could be interpreted as suggestive of lower levels of social capital, both linking and bridging. This would be consistent with the finding that Aboriginal Victorians were less likely to belong to a community group (sports, church, school, professional or other action group), also suggestive of lower levels of linking and bridging social capital.

Trust is an important indicator of social capital. Trust within social systems enables cooperative and altruistic behaviours that enhance collective wellbeing and the attainment of collective goals. Trust in our civic institutions and the people who run them, such as our healthcare system, is essential in order to maximise an individual’s health and wellbeing. We found that Aboriginal Victorians were less likely to agree that most people could be trusted or to feel valued by society. The first indicator of trust measures trust in people, essential for the creation for bonding and bridging social capital, while the second indicator measures trust in society, essential for the creation of linking social capital. Our findings of lower levels of trust among Aboriginal Victorians support the interpretation that Aboriginal Victorians have lower levels of all three types of social capital.

Racism has been shown to be a key barrier to the development of both bridging and linking social capital among Aboriginal Australians, and loss of trust has been shown to be a consequence of being a victim of racism [33,39]. Therefore, the lower levels of trust among Aboriginal Victorians may, at least in part, reflect ongoing experiences of social exclusion due to racism. Almost every Aboriginal Victorian who participated in a community survey in 2011 had experienced at least one episode of racism in the 12 months preceding the survey, and more than 70% had experienced eight or more incidents in the year preceding the survey [22]. The consequences of these experiences were that 50% of *all* participants reported high levels of psychological distress and 30% avoided various situations in daily life. Being a victim of racism has deleterious impacts on health via multiple pathways, such as mistrust leading to a reluctance to attend mainstream health services thus presenting late for medical problems, discrimination in the employment market and education system leading to higher rates of unemployment and lower educational attainment, and psychological distress which in turn can lead to mental ill-health and risk-taking behaviours [9,40].

Although our study did not directly measure experiences of racism, it could be contended that we indirectly measured experiences of racism through our measures of trust. Moreover, the 2008 VPHS did ask a question about respondents’ views on multiculturalism and found that a substantial proportion of the Victorian adult population (more than one-third) did not agree or only sometimes agreed that multiculturalism made life in their area better [41]. Therefore, we suggest that our data supports the development of policies and interventions to combat racism in Victoria as a means to promoting both bridging and linking social capital among the Aboriginal population, thereby improving health and wellbeing.

The VPHS collects limited data on a variety of health care use. With the exception of blood pressure screens and visits to a health professional for a mental health related problem, there were no differences between Aboriginal and non-Aboriginal Victorians. This could be interpreted as there being no inequalities in relation to access to health care services. However, given that Aboriginal Victorians have poorer health, one would predict that commensurate with need, Aboriginal Victorians should have received more health care services than non-Aboriginal Victorians. Therefore, it is possible that their health is negatively impacted by receiving fewer health care services than needed. This is supported by evidence that there is strong resistance by Aboriginal people to using mainstream health care services when the service fails to address cultural security, resulting in significant challenges in gaining the trust of Aboriginal clientele [42]. Moreover, there is evidence that Aboriginal people who do use mainstream health services often fail to receive the same quality of care as their non-Aboriginal counterparts. For example; Aboriginal Australians hospitalised with coronary heart disease were considerably less likely to receive key medical investigations and treatment, and those with lung or prostate cancer less likely to receive surgery [43,44].

Had we not chosen to investigate the social determinants of health, these data would have exemplified the biomedical fallacy, that is the error in inferring that the causes of disease in a population can be entirely explained by risk factors in individuals [45]. Typically, the risk factors considered and investigated are the ‘lifestyle’ risk factors, such as smoking, excessive alcohol consumption, unhealthy body weight, poor diet, and inadequate physical activity; not the psychosocial risk factors. This focus tends to ignore the fact that lifestyle risk factors have been shown to only account for approximately 26% of the total health loss due to death, disease and injury by all causes [46]. Not only did we fail to find any differences in the prevalence of the majority of lifestyle risk factors investigated, but the effect sizes that we observed for smoking, obesity and inadequate fruit intake were relatively small, compared with eight of the social determinants that we investigated. Yet the focus of most mainstream preventive policies and interventions remains around lifestyle risk factors. However, there is strong evidence that addressing psychological distress, and its causes, may be more effective in improving overall health, partly mediated by reducing the higher prevalence of lifestyle risk factors known to be associated with psychological distress, such as smoking [47]. For example, a neighbourhood renewal program in the United Kingdom, which focussed on rebuilding public housing, expectedly reduced the prevalence of mental health problems [48]. However, what was not expected was a concomitant decline in the prevalence of smoking, from 72% to 28% over a five year period, at a time when such declines across the general population were not similarly observed. Therefore, addressing potential antecedents of lifestyle risk factors, such as psychological distress, may prove to be a more effective strategy for improving overall health outcomes than focussing specifically on the lifestyle risk factors. In the case of smoking, as there has been limited success of the most commonly tried interventions of health education and social marketing, perhaps it is time to consider alternative approaches [49].

The lack of a difference in the prevalence of excessive consumption of alcohol between Aboriginal and non-Aboriginal Victorians is a particularly important finding because the negative stereotype associated with the societal belief that Aboriginal people are more likely to engage in excessive consumption of alcohol, continues to fuel prejudice and racism. Therefore, discrediting negative stereotypes is an important strategy for improving the health of Aboriginal Australians, given the association between health and experiences of racism [4]. It is also worth noting that, consistent with national findings, there was a higher prevalence of Aboriginal Victorians who abstained from alcohol consumption, and in the case of men, Aboriginal men were twice as likely to abstain as their non-Aboriginal counterparts [11].

### Strengths and limitations

To the authors’ knowledge, this is the first study of its kind in Australia that investigated the health of Aboriginal people compared directly with their non-Aboriginal counterparts using a population-based social determinants approach. It is also the first study of its kind investigating the health of Aboriginal adults in the Australian state of Victoria.

The VPHS is a population representative survey of the adult population of Victoria with a reasonable response rate of 65% in 2008; comparable to that of the 2009 U.S National Health Interview Survey (65%). Moreover, unlike the Australian national surveys, the VPHS includes both Aboriginal and non-Aboriginal adults in the same survey, allowing for direct comparison of the two populations.

There are several caveats to this study. The most important is that the data were obtained from a survey that was not designed to specifically investigate the health of the Aboriginal population. Therefore, the study did not take into consideration the significant cultural differences between the Aboriginal and non-Aboriginal populations, including the Aboriginal concept of health. Moreover, concepts such as social capital are a Western construct that may or may not be applicable to the Aboriginal population. Of perhaps greater importance, the social determinants evaluated were based on an understanding of the health of the general population, and therefore ignored crucial determinants of Aboriginal health such as experiences of racism, the impact of colonisation, connection to country, and the impact of transgenerational social processes such as the stolen generation.

The data are cross-sectional and therefore causality and its direction cannot be inferred with such a study design. The data are self-reported and therefore factors such as smoking, excessive alcohol consumption and obesity may be under-reported.

Approximately 18% of respondents refused or were unable to indicate their total annual household income, although there was no difference between Aboriginal and non-Aboriginal respondents. However, this measurement error is likely to be randomly distributed across the study population (non-differential misclassification) driving the direction of the association between the outcome and primary exposure variable towards the null [50].

The non-response analysis indicated a selection bias where males and people aged 18 to 34 years were under-represented. This was corrected by weighting the data by the sex, age and geographic distribution of the state as well as the probability of being selected. However, since the survey was conducted using computer-assisted telephone interviewing, a further selection bias was introduced by virtue of the fact that only people who could afford a landline telephone connection were included in the sample. Therefore there was an under-representation of very low SES adults. This suggests that our findings are probably highly conservative. However, this does not invalidate our findings but rather suggests that the inequalities that we observed in this study may in reality be far larger than we have been able to enumerate here.

The VPHS data were collected in 2008. However, given the considerable challenges of collecting Aboriginal data in Victoria and that very little has changed for Aboriginal people in Australia since the instigation of the ‘closing the gap’ initiative in 2007–08, we believe this data is still relevant and important [51].

## Conclusions

To summarise our key findings, we believe our work, despite the shortcomings previously discussed, goes beyond the dominant biomedical model, and demonstrates the importance of the social determinants in understanding the gap in the health of the Aboriginal compared with non-Aboriginal population. The majority of studies in the literature did not directly compare between Aboriginal and non-Aboriginal, a major strength of this work.

This is the first study of its kind in Victoria, and is suggestive of differences between Aboriginal people who reside in Victoria and Aboriginal people who reside in other states of Australia. This highlights the importance of remembering that Aboriginal people are a culturally and linguistically diverse population when interpreting the findings of the national data, as national data are likely to mask important differences.

To the best of our knowledge, this is the first population-based study in Australia that has directly measured the prevalence of depression and anxiety in an Aboriginal population. Previous studies have used psychological distress as a proxy for depression and anxiety [52].

Our findings have important implications for, and should inform, service planning and the development of evidence-based policy and interventions: namely that we need to focus on the social determinants of health that are most relevant to Aboriginal health and rethink our dependence on the biomedical model of health.

## Abbreviations

VPHS: Victorian population health survey
PR: Prevalence ratio
SES: Socioeconomic status
LGA: Local government area
BMI: Body mass index
WHO: World Health Organization
NHMRC: National health and medical research council
